# Deep Learning-Based Classification of Chest Diseases Using X-rays, CT Scans, and Cough Sound Images

**DOI:** 10.3390/diagnostics13172772

**Published:** 2023-08-26

**Authors:** Hassaan Malik, Tayyaba Anees, Ahmad Sami Al-Shamaylehs, Salman Z. Alharthi, Wajeeha Khalil, Adnan Akhunzada

**Affiliations:** 1 School of Systems and Technology, University of Management and Technology, Lahore 54770, Pakistan; f2019288004@umt.edu.pk (H.M.); tayyaba.anees@umt.edu.pk (T.A.); 2 Department of Networks and Cybersecurity, Faculty of Information Technology, Al-Ahliyya Amman University, Amman 19328, Jordan; a.alshamayleh@ammanu.edu.jo; 3 Department of Information System, College of Computers and Information Systems, Al-Lith Campus, Umm AL-Qura University, P.O. Box 7745, AL-Lith 21955, Saudi Arabia; 4 Department of Computer Science and Information Technology, University of Engineering and Technology Peshawar, Peshawar 25000, Pakistan; wajeeha.khalil@uetpeshawar.edu.pk; 5 College of Computing & IT, University of Doha for Science and Technology, Doha P.O. Box 24449, Qatar; adnan.adnan@udst.edu.qa

**Keywords:** X-rays, deep learning, CT scans, cough sound, COVID-19, lung cancer, pneumonia

## Abstract

Chest disease refers to a variety of lung disorders, including lung cancer (LC), COVID-19, pneumonia (PNEU), tuberculosis (TB), and numerous other respiratory disorders. The symptoms (i.e., fever, cough, sore throat, etc.) of these chest diseases are similar, which might mislead radiologists and health experts when classifying chest diseases. Chest X-rays (CXR), cough sounds, and computed tomography (CT) scans are utilized by researchers and doctors to identify chest diseases such as LC, COVID-19, PNEU, and TB. The objective of the work is to identify nine different types of chest diseases, including COVID-19, edema (EDE), LC, PNEU, pneumothorax (PNEUTH), normal, atelectasis (ATE), and consolidation lung (COL). Therefore, we designed a novel deep learning (DL)-based chest disease detection network (DCDD_Net) that uses a CXR, CT scans, and cough sound images for the identification of nine different types of chest diseases. The scalogram method is used to convert the cough sounds into an image. Before training the proposed DCDD_Net model, the borderline (BL) SMOTE is applied to balance the CXR, CT scans, and cough sound images of nine chest diseases. The proposed DCDD_Net model is trained and evaluated on 20 publicly available benchmark chest disease datasets of CXR, CT scan, and cough sound images. The classification performance of the DCDD_Net is compared with four baseline models, i.e., InceptionResNet-V2, EfficientNet-B0, DenseNet-201, and Xception, as well as state-of-the-art (SOTA) classifiers. The DCDD_Net achieved an accuracy of 96.67%, a precision of 96.82%, a recall of 95.76%, an F1-score of 95.61%, and an area under the curve (AUC) of 99.43%. The results reveal that DCDD_Net outperformed the other four baseline models in terms of many performance evaluation metrics. Thus, the proposed DCDD_Net model can provide significant assistance to radiologists and medical experts. Additionally, the proposed model was also shown to be resilient by statistical evaluations of the datasets using McNemar and ANOVA tests.

## 1. Introduction

Diseases that are communicable or transmissible are those that can be passed on from one person to another, as well as from one animal or insect to another [1]. These diseases are brought on by a wide variety of infectious agents, including viruses, bacteria, fungi, and others. These symptoms, however, can be rather different from one another depending on the organism that was the source of the infection [2]. The vast majority of infections do not pose a significant risk to one’s life, but some do. The life-threatening condition known as COVID-19 is caused by the severe acute respiratory syndrome coronavirus (SARS-CoV-2). In December 2019, it was discovered for the very first time in the province of Wuhan in China [1,2,3]. A pandemic was brought about as a result of the rapid and easy spread of this disease, which may be passed on from one individual to another. A healthy individual can contract COVID-19 via inhaling aerosols or droplets containing the virus; coming into direct contact with an infected person’s cough, sneeze, or voice; or breathing in droplets containing the virus [2]. If a patient is diagnosed with the illness, it is highly recommended that they self-isolate as soon as possible to prevent the virus from spreading further. The most common symptoms of COVID-19 are coughing, loss of smell, fever, lack of taste, and difficulty with breathing. Early discovery of infected individuals is crucial so that they can isolate themselves and obtain the right therapies for a quick recovery. Because the virus spreads from an infected person to those who are in close contact [4,5], early detection of infected individuals is essential.

Antigen testing, which can detect a patient who is ill at the time, and antibody testing, which can detect antibodies in the blood of a person who was previously infected with COVID-19, are used to identify a COVID-19-infected person [6]. Because the polymerase chain reaction (PCR) is used in the vast majority of antigen testing to identify COVID-19, the tests in question are referred to as PCR tests [7]. RNA is extracted from a nasal or pharyngeal swab that has been obtained as a clinical specimen to carry out this RT-PCR test [8]. Nevertheless, the processes may take a few hours; by that time, the virus may have infected a significant number of people who were previously unaffected by it [9,10]. In addition, expensive laboratory equipment and trained workers are required for PCR testing. Moreover, the sensitivity of the RT-PCR test for detecting COVID-19 is lower, which means that the test may produce a large number of false negatives. Again, a patient who has been wrongly classified as negative has the potential to contaminate a significant number of people just by associating with them [11]. It is important to establish a diagnostic system that is more reliable, has fewer instances of false negative results, and can detect the presence of COVID-19 at an early stage of infection to lessen the likelihood that it may spread [12]. Chest radiography imaging may be an alternative for fixing this issue and accelerating the identification procedure [3], as respiratory symptoms are the earliest sign of COVID-19. Both chest computed tomography (CT) scans and chest X-rays (CXRs) provide precise views of the chest’s soft tissues, bones, blood vessels, and internal organs, which is an advantage when it comes to detecting COVID-19 [6]. Furthermore, cough sounds are also utilized for identifying chest diseases [8,9,10,11,12,13,14]. A peripheral distribution, fine reticular opacity, ground-glass opacities (GGOs), diffuse distributions, bilateral involvement, and vascular thickening are some of the distinctive features that can be seen on the chest CT scan of a person infected with COVID-19 [7]. During the screening phase, great detection sensitivity for COVID-19 has been demonstrated by both CT and CXR [8,9]. On the other hand, radiologists may experience visual tiredness, which might hinder them from diagnosing certain small lesions [10,11,12]. Because of the current situation, it is necessary to use computerized diagnosis that is based on artificial intelligence (AI) for the diagnosis of COVID-19 and other chest diseases.

The death rates are rising to frightening levels, but if patients are detected and treated quickly, their chances of surviving are greater than 95%. Because of this, we are motivated to create a novel method for the identification of nine different types of chest diseases, including COVID-19, edema (EDE), lung cancer (LC), pneumonia (PNEU), pneumothorax (PNEUTH), normal, atelectasis (ATE), and consolidation lung (COL) to save human lives. In this paper, we present a novel multi-classification model, called the deep learning (DL)-based chest disease detection network (DCDD_Net), which uses a CXR, CT scans, and cough sound images to identify nine different chest diseases. Most research studies [1,5,8,12,13,14,15] have indicated great performance in binary classification, i.e., differentiating between COVID-19 and healthy cases. However, no evidence has been found of using DL models for the identification of nine different types of chest diseases, including COVID-19, EDE, LC, PNEU, PNEUT, ATE, COL, and normal using CXR, CT scans, and cough sound images. The proposed DCDD_Net model was trained on 20 publicly available benchmark datasets of CXR, CT scans, and cough sound datasets. The scalogram method was applied to convert the cough sound into a cough sound spectrogram image. Additionally, DCDD_Net was also compared with four baseline classifiers: InceptionResNet-V2, EfficientNet-B0, DenseNet-201, and Xception. The major contributions of this study are presented below:

1. The novel DCDD_Net model that is suggested is intended to diagnose each of the nine distinct forms of chest disease. The model that is proposed can extract dominating features from CXT, CT scans, and cough sound images, which can be of assistance in providing an accurate diagnosis of chest diseases.
2. The scalogram method was used to convert the cough sounds into an image.
3. For this work, we simplified the model by cutting down on the total number of trainable parameters to produce a reliable classifier.
4. As a result of the issue of class imbalance that exists in CXT, CT scans, and cough sound image datasets, the accuracy of the DCDD_Net model was significantly reduced. We circumvented this problem by employing an upsampling strategy known as BL-SMOTE, which allowed us to collect mixture samples of the image at each class to achieve greater accuracy.
5. The suggested DCDD_Net model achieved superior results in comparison to four baseline classifiers, namely, InceptionResNet-V2, EfficientNet-B0, DenseNet-201, and Xception, in terms of numerous assessment measures, including accuracy, area under the curve (AUC), precision, recall, loss, and F1 score.
6. Additionally, when compared to the most recent state-of-the-art (SOTA) classifiers, the suggested DCDD_Net model provided results that were both significant and notable.

## 2. Literature Review

A significant number of studies on the diagnosis of chest diseases have been carried out to help medical experts identify the disease from the beginning. On the contrary, recent studies have concentrated on the creation of various AI techniques that can automate the detection of various kinds of chest diseases. The most recent studies on the diagnosis of chest diseases using DL models are summarized in Table 1.

### 2.1. Deep Learning Models for Chest Disease Classification Using Chest X-rays and CT Scans

Iqbal et al. [13] introduced TBXNet, a DL network that is easy to use and very effective. It was able to categorize a very large number of TB images by utilizing CXR. Furthermore, data that had been trained before were transferred to the fusion layer via the pre-trained layer. The accuracy of the proposed TBXNet was measured at 98.98% on Dataset A and 99.17% on Dataset B. Validation of the generalizability of the proposed study was accomplished by utilizing Dataset C, which consisted of imaging data from CXR that were either normal, TB, PNE, or COVID-19, and it obtained 95.10% accuracy. By applying images obtained from chest X-rays, Kumar et al. [14] utilized an ensemble model that was able to identify COVID-19 at the earliest stage of the disease. Ensemble learning was utilized throughout the process of developing the suggested model. Three transfer learning models were specifically added to the process: GoogLeNet, EfficientNet, and XceptionNet. Patients were categorized as having COVID-19, PNEU, or TB or as being healthy according to these models. The generalization capacity of the classifier was improved by the model that is proposed, and this improvement was applied to both binary and multiple-class COVID-19 datasets. The effectiveness of the proposed ensemble model was assessed through the utilization of two well-known datasets.

The CBAMWDnet model was utilized by Huy et al. [15] to identify TB in an image of a chest X-ray. The model was built using the convolutional block attention module (CBAM) and the wide dense net (WDnet) structure, both of which were intended to successfully capture visual and contextual elements within images. In terms of accuracy, the proposed model outperformed the other models by 98.80%. The COVID-CheXNet system was developed by Al-Waisy et al. [16] to detect COVID-19 in chest X-ray pictures. This system uses a hybrid DL architecture. First, the brightness of the X-ray image was improved using the CLAHE method, and the noise level was reduced using a Butterworth bandpass filter. After that, two discriminating DL algorithms, ResNet-34 and HRNet, were developed on the pre-processed CXR images to strengthen the most recently developed model’s generalization skills and prevent overfitting. The efficacy of the COVID-CheXNet system was evaluated by generating a large-scale dataset of X-ray images called the COVID-19 vs. normal database.

Malik et al. [17] developed and evaluated a multi-classification strategy that relies on the DL model for automatically recognizing LC, PNEUTH, COVID-19, TB, and PNEU from CXR pictures. The CNN model known as CDC Net, which uses residual network perception and dilated convolution, was applied to identify COVID-19 and other conditions affecting the respiratory system. When recognizing various chest disorders, CDC Net achieved an AUC of 0.9953, with an accuracy of 99.39%, a precision of 99.4%, and a recall of 98.13%.

A classification approach that can evaluate CXR and help with the precise identification of COVID-19 was proposed by Shelke et al. [18]. The CXR images obtained using their approach were divided into the following four groups: normal, TB, PNEU, and COVID-19. VGG-16 was the DL model used to categorize PNEU, TB, and normal, with a test accuracy of 95.9%. DenseNet-161 was used to differentiate between normal, PNEU, and COVID-19, with a test accuracy of 98.9%, but ResNet-18 performed well in severity categorization, with a test accuracy as high as 75%. Their method enables the screening of huge populations because it relies heavily on X-rays as a key testing component for COVID-19.

By applying CXR as their primary data source, Ali et al. [19] developed a 19-layer CNN model to detect chest infections. The developed model was then reapplied to identify various kinds of chest infections using transfer learning. These included COVID-19, fibrosis, PNEU, and TB. The model was improved by the use of a stochastic descent of gradients with momentum. The proposed multiple-phase structure achieved a classification accuracy of 98.85% for online CXR datasets for detecting chest infections. The accuracy of the proposed multiple-phase CNN approach was further confirmed by employing an additional dataset, which revealed a 98.5% level of accuracy.

Constantinou et al. [20] identified COVID-19 using DenseNet-121, DenseNet-169, ResNet-50, ResNet-101, and Inception-V3 with transfer learning. The most extensive archive of COVID-19 CXR pictures that were available to the public was used during the development and verification of all of the models. There were 11,956 images of patients who had been confirmed to have COVID-19, 11,263 images of patients who had viral or bacterial pneumonia, and 10,701 images of healthy individuals. The ResNet-101 model had the best overall performance, scoring 96% in each of the categories measuring accuracy, precision, and recall. Performance levels for the remaining models were all satisfactory.

Agrawal et al. [21] focused on identifying COVID-19 from CXR pictures by exploring a binary categorization such as COVID-19 vs. non-COVID-19 and classification with multiple classes such as COVID-19, non-COVID-19, and PNEU. The dataset was made up of 125 CXR images for COVID-19, 500 CXR images for no findings, and 500 CXR images for pneumonia. They tested and evaluated a variety of DL models, including VGG19, InceptionV3, ResNet50, MobileNetV2, DenseNet121, and Xception, in addition to specialized models such as DarkCOVIDNet and COVID-Net, and they found that ResNet50 performed most effectively out of all of them. To classify COVID-19, non-COVID-19, bacterial PNEU, viral PNEU, and normal CXR images obtained from a variety of publicly accessible sources, Ibrahim et al. [22] recommended the development of a DL technique that made use of a pretrained AlexNet algorithm. The model’s accuracy was 93.42%, its sensitivity was 89.1%, and its specificity was 98.92%.

Ayalew et al. [23] introduced a reliable approach for classifying CXR images as those of normal vs. COVID-19 patients. This model was constructed using CNN, dropout, batch normalization, activation function, and Keras parameters. The images were subsequently categorized into a predefined class (normal vs. COVID-19) by utilizing the knowledge gained from the learning process model and SVM. The findings of the research reveal that each of the models generated favorable outcomes, with picture segmentation, augmentation, and image cropping providing the most successful outcomes, with a test accuracy of 99.8%.

Jennifer et al. [24] evaluated various deep learning models, such as ResNet-50, VGG-16, and XGBoost, for COVID-19 classification using a neutrosophic set approach. They achieved a remarkable classification accuracy of 97.33%. Jaszcz et al. [25] proposed a heuristic red fox optimization algorithm (RFOA) for medical image segmentation. Their proposed model achieved a classification accuracy of 97.20% and 94.35% for the Jaccard index. Karthik et al. [26] focused primarily on the most recent advances in image-based COVID-19 detection methods that involve classification and segmentation. By using edge-supervised information in the first stage of downsampling, Hu et al. [27] created a model edge supervised module (ESM) to emphasize low-level boundary features. The mask-supervised information can be integrated into the following step, where an auxiliary semantic supervised module (ASSM) is proposed to improve the quality of high-level semantic information. The semantic gaps between high-level and low-level feature maps are then reduced by adding an attention fusion module (AFM) to fuse various scale feature maps of different levels. Their findings demonstrate that the three proposed modules were effective at raising the dice metric by 1.12%. A unique prior knowledge-based algorithm for assessing the severity of COVID-19 was created by Li et al. [28] by utilizing CT scan images. They were successful in mining the result with an accuracy of 86.70%.

### 2.2. Deep Learning Models for Chest Disease Classification Using Cough Sounds

Pahar et al. [29] introduced an automated cough classifier that was created using DL. This classifier was able to differentiate between TB, COVID-19, and healthy cough sounds. The cough recordings were taken in a variety of situations, including indoors and outdoors, and were provided through the use of smartphones by people located all over the world; consequently, they contained varied degrees of background noise. CNN, LSTM, and Resnet50 were trained and evaluated using 1.68 h of TB cough sounds, 1.69 h of healthy cough sounds, and 18.54 min of COVID-19 cough sounds from 47 patients with TB, 1498 healthy patients, and 229 patients with COVID-19, respectively. Kim et al. [30] proposed MFCC, -MFCC, 2-MFCC, and wavelength contrast as a characteristic set designed for the identification of COVID-19 and implemented it in an algorithm that incorporates DNN and ResNet-50. The Coswara, Cambridge, and COUGHVID crowdsourcing databases provided them with the cough sound data that were used in their research. After the development of both the ResNet-50 and the DNN models, the respective values for accuracy, sensitivity, and specificity were 0.96, 0.95, and 0.96. Using this approach, an Android application for COVID-19 testing was created so that a large number of individuals could utilize it.

Islam et al. [31] created a research study containing the development of an algorithm for the noninvasive and automatic identification of COVID-19 by employing cough audio recordings and DNN. The noises generated by coughing can provide important information regarding the movement of the glottis in several different respiratory disorders. By applying cough audio recordings taken from healthy individuals and those with COVID-19 infections, the efficacy of the proposed algorithm was assessed. The proposed technique automatically recognizes COVID-19 cough audio recordings with a total accuracy of 89.2%, 93.8%, and 97.5%, while using time-domain, mixed-domain, and frequency-domain vectors of features, respectively.

Loey et al. [32] were able to identify and categorize characteristics by employing a total of six different deep transfer models. These models were ResNet-18, ResNet-50, GoogleNet, ResNet-101, NasNetmobile, and MobileNet-V2. The database contains a total of 1457 different cough sounds, 755 of which are from COVID-19 and 702 from healthy people. The SGDM optimizer discovered that the accuracy of the proposed model was 94.9%. The phase of sound-to-image conversion was improved through the scalogram method.

Nessiem et al. [33] assessed the use of DL models as a pervasive, affordable, and high-performing pre-testing approach for recognizing COVID-19 from recorded sounds of respiration or coughing obtained on mobile devices via the internet. They employed an ensemble of CNNs that can determine whether an individual has been impacted by COVID-19 based on the audio of raw breathing and coughing as well as spectrograms. Their proposed models were able to achieve a maximum UAR value of 74.9% and an AUC value of 80% in the held-out individual independent evaluation division. Tawfik et al. [34] developed a smart strategy that made use of DL to identify COVID-19 patients by listening to patients’ cough sounds. Their system consisted of three distinct phases: sound processing before use through noise reduction; the extraction of features, segmentation, and categorization; and the implementation of models. A total of 1635 audio subjects were analyzed, and 8 features were identified from those recordings. A total of 573 coughs tested positive for COVID-19, whereas 1062 coughs tested negative for the virus. In terms of detecting COVID-19, the DL model had an overall accuracy rate of 98.5%.

CBIR-CSNN was proposed as a method to differentiate between LC and TB in CT images by Zhang et al. [35]. Initially, the lesion regions were clipped out to generate the LC and TB databases, and then pairs of two different places were used to generate the patch–pair database. CBIR-CSNN was trained and tested on a total of 719 patients who were used throughout the process. To validate CBIR-CSNN, an additional external dataset with 30 patients was utilized. At the patch level, the CBIR-CSNN achieved remarkable results of 0.953 maP, 0.947 accuracy, and 0.970 AUC value. Multi-scale blocks of residual networks and open dense connections are the two components that make up the DAvoU-Net model that was proposed by Alebiosu et al. [36]. This model is used to divide TB-affected regions based on CT scans. The feature learning approach initiates a three-dimensional CNN for the deep extraction of features by transforming the two-dimensional values of a well-trained NN into three-dimensional values. In general, the overall performance of DAvoU-Net + ResNet-50, a 3D CNN, and a simultaneous LSTM was superior to that of the other six fully trained NNs that were used for comparison.

Toaçar et al. [37] introduced a method to detect lung cancers by using chest CT scans. The AlexNet, LeNet, and VGG-16 DL algorithms were utilized for the extraction of features and categorization. During the training of the models, image augmentation techniques such as zooming, rotation, filling, and cropping were implemented in the dataset to improve the categorization success rate. Due to the remarkable efficacy of the model, the features that were acquired from the final FCL of the AlexNet framework were used independently as inputs to LR, LDA, decision tree, SVM, SoftMax, and KNN classifiers. The combined use of the AlexNet algorithm and the kNN classifier provided the highest accuracy in classification at 98.74%.

Latif et al. [38] proposed the use of DL techniques to extract features. These algorithms were GoogleNet and ResNet-50. When integrating GoogleNet, ResNet18, and the SVM method in conjunction with the modified ML process, the maximum average accuracy that could be achieved was 99.9% after 2000 features were generated. P-DenseCOVNet is a modified version of the DenseNet structure that was designed by Sadik et al. [39] for the effective extraction of features and the evaluation of COVID-19 and pneumonia. In this structure, direct convolutional paths were added to the standard DenseNet method to improve achievement by overcoming the loss of spatial conflicts. To successfully segment the lung regions from CT scans, an upgraded version of U-Net known as SKICU-Net, containing skip connections among the decoder and encoder sections, was applied rather than the conventional U-Net. This resulted in a superior segmentation performance. A high level of achievement was shown by the system, which received a 0.97 F1-score for the task of segmenting and achieved an 87.5% accuracy when identifying normal cases, COVID-19, and common pneumonia. A federated learning method for the detection of COVID-19 using previous training DL methods was proposed by Florescu et al. [40]. In their study, a total of 2230 central CT scans of the chest were collected, including 1016 images of COVID-19, 610 images of LC, and 604 normal images. The architecture concept consisted of a single server and three clients. Each client had a collection of data. A healthcare organization that possessed a private dataset represented a client. These organizations worked together to develop a global model.

A diagnostic tool based on AI categorization of chest CT scans was created by Fu et al. [41] to diagnose COVID-19 and other prevalent infectious respiratory diseases. A total of five lung conditions were evaluated, and they were as follows: COVID-19, bacterial PNEU, viral PNEU, TB, and normal lung. Images of the training and validation groups were gathered at Wuhan Jin Hospital. Images of the test group were taken at Xiamen University and Zhongshan Hospital. The efficiency of the proposed AI system was impressive when it came to recognizing COVID-19 and other frequent viral respiratory diseases with equivalent levels of recall and specificity. Kaewlek et al. [42] tested four DL models, which included GoogleNet, ResNet, AlexNet, and deep CNN, for categorizing CT scans of TB, PNEU, and COVID-19. They obtained 2134 photos of normal cases, 943 images of TB, 2041 images of PNEU, and 3917 images of COVID-19 from internet sources. According to the results of their analysis of the effectiveness of the model, ResNet had the highest accuracy at 0.96, a 0.93 F1 score, and an AUC score of 0.95 AUC. The model with the second-greatest result was DCNN, followed by AlexNet and GoogleNet in that order. A deep CNN-based technique developed by Polat et al. [43] was capable of independently recognizing patterns associated with COVID-19-related lesions in chest CT images. Originally, 102 CT scans were segmented, which resulted in the production of a total of 16,040 CT scan segments. After that, 10,420 CT scan segments that corresponded to healthy respiratory areas were recognized as COVID-19-negative, whereas 5620 CT scan segments in which various lesions had been discovered were identified as COVID-19-positive. The accuracy of the diagnosis was able to be raised to 93.26% by utilizing the CNN architecture that was suggested.

Abayomi-Alli et al. [44] proposed a DL model called DeepShufNet for COVID-19 detection. Using the Mel COCOA-2-augmented training datasets, the suggested model had an accuracy of 90.1%, a precision of 77.1%, a recall of 62.7%, a specificity of 95.98%, and an f-score of 69.1% for identifying cases of COVID-19.

Mishra et al. [45] developed an algorithm for identifying COVID-19 from CT images that includes COVID-19, normal, and PNEU groups using their transfer learning method, which relies on the ResNet50 and VGG-16 architectures. Their research employed data enhancement and fine-tuning methods to enhance and optimize the ResNet50 and VGG16 algorithms. With a standard classification accuracy of above 99.9% for both ResNet-50- and VGG-16-based systems, the model that was suggested works extremely well for binary classification tasks such as comparing COVID-19 to normal. In the classification of multiple classes, such as COVID-19 vs. normal vs. pneumonia, the suggested approach achieved a median accuracy of classification of 86.74% and 88.52% when utilizing the VGG16 and ResNet50 architectures as the initial state, respectively. Masud et al. [46] developed a diagnostic strategy based on CNN to identify COVID-19 patients by evaluating the picture properties of CT scans. To identify COVID-19-infected individuals, their research examined a freely accessible CT scan database and inputted it into the suggested CNN approach. There were 5493 non-COVID-19 photos and 3914 images with COVID-19 in the CT scan database. During the training, validation, and evaluation stages of its development, the model achieved an accuracy of 99.76%, 96.10%, and 96%, respectively.

**Table 1:** A list of previous studies that used ML and DL models for the diagnosis of chest diseases using CXR, CT scans, and cough sounds.

Reference	Year	Models	Diseases	Types	Accuracy	Strength	Weakness
[15]	2023	CBAMWDnet	TB and normal	CXR	98.80%	The model was suitable for TB and normal case classification using CXR.	The model was trained and tested on imbalanced datasets.
[16]	2023	COVID-CheXNET	COVID-19 and normal	CXR	92.99%	The model was trained on the chest X-ray dataset and achieved remarkable results in classifying COVID-19 patients.	No augmentation method was used and datasets required an image enhancement process due to the poor quality of CXR.
[17]	2023	CDC_Net	COVID-19, PNEUTH, PNEU, LC, and TB	CXR	90.39%	The model could classify five different chest diseases.	There was a gradient-boosting issue.
[19]	2023	CNN	COVID-19, fibrosis, and TB	CXR	93.85%	The model was appropriate for classifying COVID-19, TB, and fibrosis using CXR.	Pre-processing of the dataset was not performed.
[20]	2023	ResNet-50, ResNet-101, ResNet-121, DenseNet-169, and Inception-V3	COVID-19, non- COVID-19 (viral and bacterial PNE) and normal	CXR	96.6%	Different pre-trained models were used for evaluating the COVID-19 cases.	Even having a very extensive ResNet did not ensure that all residual blocks would be included in the operations.
[21]	2023	VGG-19, ResNet-50, MobileNet-V2, Inception-V3, Xception, DenseNet-121, Dark COVIDNet, and COVID-Net	COVID-19, non- COVID-19, and PNEU	CXR	86.13%	Several transfer learning models were used to identify COVID-19 and pneumonia-infected CXR.	The datasets were imbalanced.
[23]	2023	DCNN	COVID-19 and normal	CXR	99.10%	A deep-layer network model was designed for COVID-19 classification.	The model was trained and tested on very few image samples.
[30]	2023	ResNet-50 and DNN	COVID-19 and healthy	Cough Sound	96.00%	A neural network and a pre-trained model were used to identify COVID-19 using cough sound images.	No noise removal method was applied.
[36]	2023	DAvoU-Net + ResNet-50	TB and normal	CT scan	81.19%	Ensembling of DavoU-Net + ResNet-50 was used for image segmentation and classification of TB and normal.	The study did not focus on the CT scan slices.
[42]	2023	GoogleNet, AlexNet, ResNet, and DCNN	PNEU, TB, and COVID-19	CT scan	96.6%	Several well-renowned models were tested for the identification of pneumonia, TB, and COVID-19.	The datasets were imbalanced.
[13]	2022	TBXNet	COVID-19, normal, PNEU, and TB	CXR	95.10%	A significant TBXNet was developed for TB case classification.	The datasets were imbalanced.
[29]	2022	CNN, LSTM, and ResNet-50	TB, COVID-19, and healthy	Cough Sound	92.59%	A concoction of CNN with LSTM and a pre-trained model were used to find TB and COVID-19 disease classification.	LSTMs are prone to overfitting and it was difficult to apply the dropout algorithm to curb this issue.
[31]	2022	DNN	COVID-19 and healthy	Cough Sound	97.5%	A deep neural network model was used for COVID-19 using cough sounds.	There was a gradient-boosting issue.
[34]	2022	CNN	COVID-19 and non-COVID-19	Cough Sound	98.50%	A CNN-based model was designed for COVID-19 cases.	There was an increasing gradient and overfitting problem.
[35]	2022	DL + CBIR	LC and TB	CT scan	94.7%	A combination of DL with CBIR was used to extract significant information from CT scans for LC and TB case classification.	A semantic gap existed that may have affected the classification performance.
[38]	2022	GoogleNet + ResNet-50	COVID-19, PNEU, and normal	CT scan	99.9%	A combination of two transfer learning models was used for COVID-19, PNEU, and normal cases.	Data validation was not performed.
[39]	2022	P-DenseCOVNet	COVID-19, PNEU, and normal	CT scan	87.51%	A dense network was developed for COVID-19, PNEU, and normal classification.	There was a gradient-boosting issue.
[40]	2022	Federate Learning VGG-16	COVID-19, LC, and normal	CT scan	79.32%	A secure model was designed for data sharing.	Disease classification was not focused on.
[12]	2021	EfficientNet, GoogleNet, and XceptionNet	COVID-19, PNEU, and TB	CXR	99.21%	Pre-trained models were used for lung disease classification.	There was a lack of interpretability.
[18]	2021	DenseNet-101, VGG-16, and ResNet-18	COVID-19, PNEU, normal, and TB	CXR	98.90%	A deep-layered model was designed for COVID-19 cases.	The models were trained and tested on a limited dataset.
[22]	2021	AlexNet	COVID-19, non- COVID-19 (viral and bacterial PNE), and healthy	CXR	93.42%	The proposed model was designed for bacterial and viral pneumonia.	The datasets were imbalanced.
[32]	2021	ResNet-18, GoogleNet, ResNet-50, ResNet-101, MobileNetV2, and NasNetMobile	COVID-19 and healthy	Cough Sound	94.90%	Several pre-trained models were tested to discover COVID-19 cases using cough sounds.	No noise removal methods were used. The time frame of the cough sounds was not considered.
[33]	2021	CNN	COVID-19 vs. non-COVID-19	Cough Sound	74.9%	A simple CNN model was used for COVID-19 classification using sounds.	No pre-processing methods were used.
[43]	2021	DCNN	COVID-positive and COVID-negative	CT scan	93.24%	A deep network was developed for COVID-19 cases.	A very limited dataset was used.
[45]	2021	VGG-16 and ResNet-50	COVID-19, PNEU, and normal	CT scan	88.52%	VGG-16 and ResNet-50 were integrated for COVID-19 using CT scan.	CT scan images were not pre-processed before being applied to training the model.
[46]	2021	CNN	COVID-19 vs. non-COVID-19	CT scan	96%	A 6-layer CNN model was developed for lung disease classification.	Few image samples were used.
[37]	2020	AlexNet + KNN	LC and normal	CT scan	98.74%	The proposed model was combined with KNN for lung cancer classification.	The normal class had more images than the LC class, which affected the model performance.
[41]	2020	AI	COVID-19, PNEU, TB, and normal	CT scan	99.4%	A computer-assisted model was developed for several chest diseases.	There was a lack of training data, imbalanced data, and interpretability of data.

According to many studies [14,15,16,17,18,19,20], the symptoms of nine different chest diseases, i.e., LC, ATE, COL, TB, PNET, EDE, COVID-19, PNEU, and normal, are similar to each other. It is a challenge for health experts to identify these chest diseases using CXR and CT scans. Similarly, healthcare professionals have also attempted to diagnose these chest diseases using cough sounds [29,31,32,33,34]. However, cough sounds also resemble each other among these diseases. Therefore, it is also a challenge for health experts to diagnose chest diseases based on cough sounds. Hence, there is an evident need to develop an automated framework based on DL models that can automatically diagnose chest diseases as mentioned above using X-rays, CT scans, and cough sounds. The main focus of previous studies [30,31,32,33,34,35,38] was to diagnose COVID-19 and non-COVID-19 cases from CXR images and CT scans. A few research studies [29,30,31] have employed the use of CXR images to identify COVID-19 from pneumonia infections, including viral and bacterial infections. However, limited studies [41,42,43,44,45,46] have identified PNEU and COVID-19 based on cough sounds, and no evidence has been found to diagnose LC, ATE, COL, TB, PNEUTH, and EDE based on cough sounds using DL models. Therefore, to overcome the challenges mentioned above, this research study developed a DL framework that can detect multiple chest diseases based on X-ray images, CT scans, and cough sound images.

## 3. Materials and Methods

This section describes the experimental approach that was used to evaluate the effectiveness of the model that was proposed, as well as four widely recognized deep CNN classifiers, namely, InceptionResNet-V2, EfficientNet-B0, DenseNet-201, and Xception.

### 3.1. Proposed Model for the Diagnosis of Chest Diseases

In the field of healthcare and medicine, image processing has created a revolution. It is used in virtually every area of healthcare nowadays, particularly in the pre-analysis stage [47,48,49]. During the diagnostic phase, doctors may check the internal organs of an individual without the need for an operation. In the medical sector, there is a variety of scans, including X-ray and computer tomography (CT) scans. A medical expert is incapable of analyzing medical imaging accurately because it takes a significant amount of time. A computer can derive accurate conclusions from them because a machine that was trained on a database of health-related picture data can provide precise results in a matter of seconds [50,51,52]. The research community plays an essential role in the creation of sophisticated automated systems for accurate and rapid assessments and supports the enhancement of these systems daily [53,54,55].

**Figure 1:**
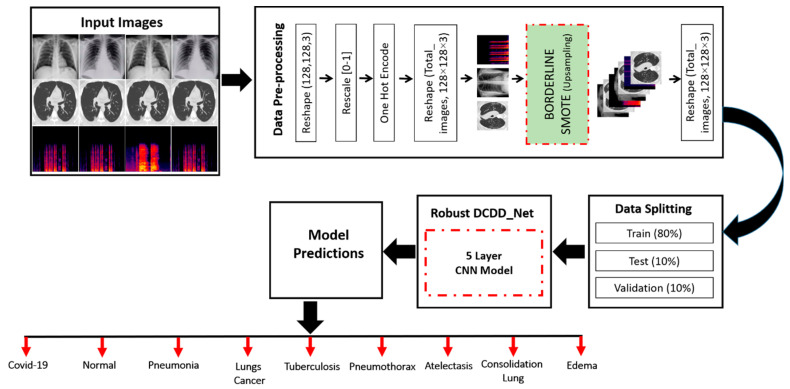
Workflow of the proposed DCDD_Net.

In this study, we developed a novel deep learning-based chest disease detection network (DCDD_Net) that uses a CNN. This model was trained and evaluated using images of nine major chest disease categories, including ATE, COL, COVID-19, EDE, PNEUTH, normal, PNEU, LC, and TB. The size of the input image was specified as 128 × 128 pixels. The dataset of images was pre-processed by normalization, and the critical phase of modifying the data with categorical variables was provided to the proposed DCDD_Net. Then, we used the borderline synthetic minority oversampling technique (BL-SMOTE) to balance the number of samples in each class and resolve the issue of a dataset that is imbalanced. The chest disease dataset was categorized into three separate groups: testing, training, and validation. In addition, Figure 1 illustrates the workflow of the proposed DCDD_Net for the identification of chest diseases. The study’s experiment was conducted for no longer than 40 epochs. As soon as all of the epochs had passed, the proposed DCDD_Net reached the accuracy level that had been anticipated throughout the training and validation processes. The effectiveness of the proposed method (DCDD_Net) was compared to that of four pre-trained models using the following metrics: accuracy, recall, loss, AUC, precision, and F1-score.

### 3.2. Dataset Description

This section is further separated into two subsections. The first section provides multiple CXR and CT scan image databases for chest diseases. The remaining section defines cough sound datasets associated with chest diseases.

#### 3.2.1. Dataset of CXR and CT Scan Images for Chest Diseases

**Figure 2:**
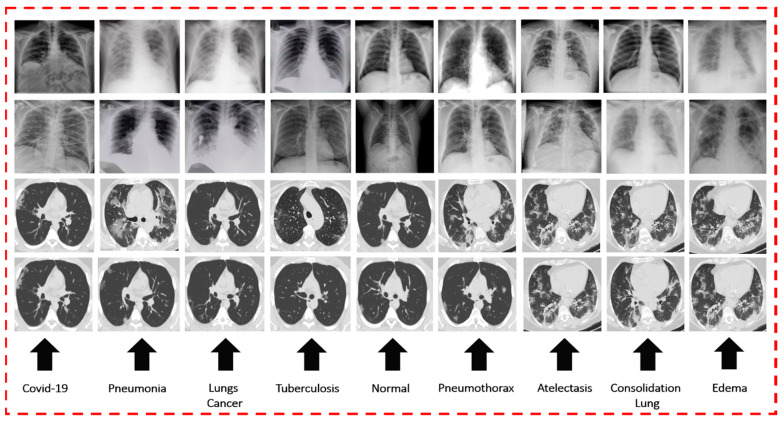
CT scan and CXR sample images of nine chest diseases.

For training and verifying the models of DL via CXR, seven publicly accessible datasets on a variety of chest diseases were obtained from a large number of different sources. Initially, we gathered 423 chest radiographs of COVID-19 infections from Mendeley [56] and GitHub [57] sources. The chest radiographs of normal or healthy individuals were obtained from two datasets, namely, NIH [58] and Kaggle [59] chest radiographs. The images of pneumonia were obtained from the RSNA [60]. These datasets include 247 images of normal X-rays and 189 images of pneumonia X-rays. A total of 931 X-ray images were collected from the NIH [61], which were categorized as follows: 425 images of PNEUTH, 154 images of ATE, 198 images of EDE, and 154 images of COL. The remaining CXR images from the NIH dataset were excluded from this study. The dataset of lung cancer was taken from [62], and 74 CXR images were obtained from the dataset. Last, a total of 259 CXR images of patients diagnosed with TB were collected [63]. Figure 2 shows a sample image of COVID-19 as well as other chest diseases on CXR and CT scans.

For training and verifying the proposed DCDD_Net via CT scans, seven publicly accessible datasets on a variety of chest diseases were obtained from a large number of different sources. There was a total of 426 positive chest CT scans for COVID-19 that were taken from reference [64]. A total of 118 LC images from CT scans were gathered from the freely accessible dataset referred to in [63]. Sources [65,66] were used to obtain CT scan images of various chest diseases, such as COL, EDE, PNEUTH, and ATE. The dataset includes a total of 580 images, such as 12 images of COL, 217 images of ATE, 160 images of PNEUTH, and 91 images of EDE. We obtained a total of 168 images from CT scans of pneumonia [67]. We recovered 112 TB images of CT scans by utilizing the open-source database provided in [68]. A total of 672 CT scan images of normal people were obtained from [69].

#### 3.2.2. Dataset of Cough Sounds for Chest Diseases

For training and evaluating the proposed DCDD_Net, various cough sound databases were gathered. The Coswara database, which is open to the public, was used to collect a total of 310 cough sounds, including the sounds of 100 COVID-19-positive patients and 210 healthy individuals [70]. The objective of the Coswara project is to create a COVID-19 detection instrument based on respiratory system sounds and coughing [71]. Participants were instructed to submit audio of their coughing into an internet-based data collection instrument that could be retrieved through their smartphones. The sound data that were collected included a combination of shallow and deep coughing, rapid and unsteady breathing, broadened vowel phonation, and spoken numbers. Additionally, the patient’s gender, year of birth, place of residence, present health status, and previous health issues were documented. The recorded sound frequency was 44.1 kHz, and all regions besides Africa were represented in the audio sample set. We obtained a total of 292 cough sounds from TB patients [72]. The Respiratory Audio Database was created by a pair of research groups from Portugal and Greece [73]. It includes 920 labeled samples that vary from 10 to 90 s in length. It contains a total of 5.5 h of sound recordings that involve 6898 breathing phases, 886 of which contain wheezes, 1864 of which contain crackles, and 506 of which contain both of them. The data include recordings of both soft and harsh breathing sounds that simulate environments in the real world. There are 119 sounds of coughing related to pneumonia, 90 sounds of coughing linked to ATE, 80 cough sounds related to COL, 39 coughing sounds related to edema, and 42 cough sounds linked to pneumothorax in the dataset. In the end, 222 sounds of coughing from LC patients were gathered [74]. Table 2 provides statistics on the cough audio databases.

### 3.3. Conversion of Cough Audio to an Image

**Figure 3:**
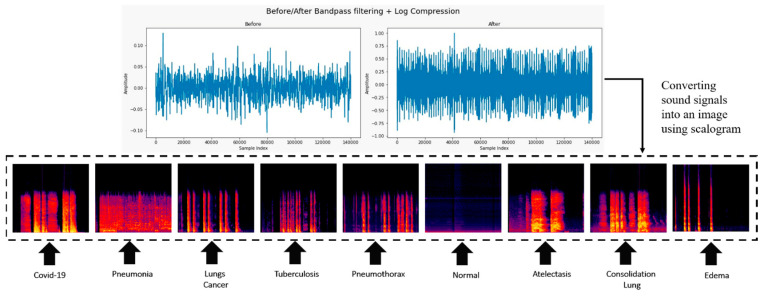
Scalogram images of nine cough sounds of chest diseases.

Scalograms represent the actual frequencies of a wave’s continuous wavelet transform (CWT) factors [75]. For both of the measurements that were taken in this study, the scalogram method was utilized. At first, the noise reduction process was applied to the one-dimensional sound of coughing in the various chest disorder datasets. Second, two-dimensional scalograms based on CWT were added to the preprocessed signals. Cough signals utilize CWT to convey data from the time domain to the frequency domain, as demonstrated in Figure 3. Convolution is a successful method for removing both high- and low-frequency sounds, particularly when used in conjunction with a bandpass filter. Using the wave’s internal components, the CWT, which is comparable to the Fourier transform, identifies the degree of similarity between a mathematical function and a wave. The CWT of the formula T(S) on a scale (a > 0) is determined using Equation (1). The function that represents the father signal, denoted by (S), is constant throughout the frequency and time domains. The values of the constantly varying dimension parameter are denoted by a, whereas the position parameter is denoted by b. The coefficients of the CWT method produce a series of wavelets that are ordered according to scale and location. The role of the father signal is to deliver the generational root characteristic that the children’s signals require to function correctly. CWT generates the cough audio signal by combining the scale parameter with the father signal [75,76,77].

(1) $$CWT\;\left(a,b\right)=\frac{1}{{\left|a\right|}^{0.5}}\int_{-\infty }^{\infty }T\left(S\right)\;\theta (\frac{s-b}{a})ds$$

The following steps were involved in converting cough sounds into images.

1. We collected several different types of cough sound image databases.
2. All cough sound recordings had the same sampling rate, such as 44.1 kHz.
3. A low-band pass filter method was used to remove the unwanted background noise.
4. The CWT method was applied to convert a cough sound signal into its frequency domain representation over time.
5. In a scalogram, the scale of the frequency axis changes with time.
6. The scalogram transformation is a 2D matrix, where one axis represents time and another axis represents frequency.
7. We mapped the intensity values to colors by using a heatmap color map and created an image-like representation of the cough sound signal’s frequency content over time.

### 3.4. Using BL-SMOTE to Balance the Class’s Samples

To tackle the problem of unequal class representation in the dataset, we referred to the upsampling methodology. Upsampling is when more samples with zero values are inserted between each of the original samples so that the sampling rate can be increased. To produce fusion data for each category, this method makes use of the upsampling strategy known as BL-SMOTE [78]. In this method, the classification process begins with the analysis of the minority class. If every neighbor belongs to the majority class, it classifies every minority data point as a noise point and dismisses it when synthesizing synthetic data [79]. Furthermore, it resamples exclusively from a limited number of border neighborhoods that belong to both minority and majority groups [80]. Table 3 depicts the arrangement of samples before the start of the upsampling process. The order in which the samples were distributed can be seen in Table 4, which was generated after upsampling was performed.

### 3.5. Proposed Model

The next section describes the proposed DCDD_Net and its architecture for the classification of chest diseases.

#### 3.5.1. Detailed Structure of the Proposed DCDD_Net

**Figure 4:**
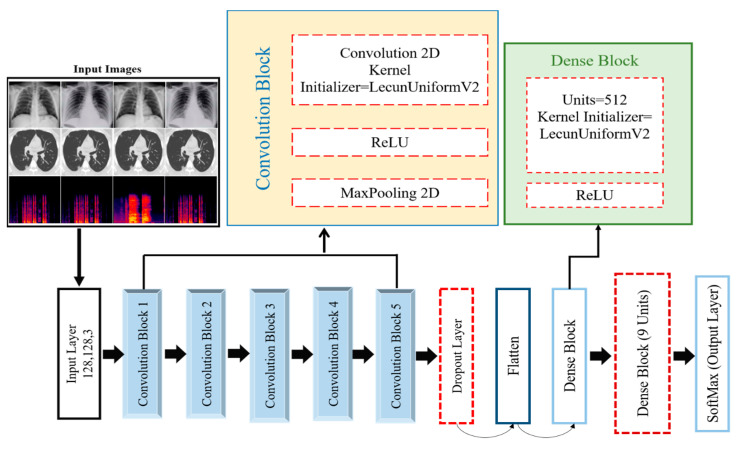
The architecture of the proposed DCDD_Net to identify chest diseases.

CNN architecture depends on the biological framework of the brain of humans and is primarily employed in computer vision applications such as the classification of images, identification of objects, and image segmentation. It was preferred for recently developed deep models because of its translational invariance [81]. Translation invariance signifies that a CNN can identify the same feature, no matter its position in different images. In this research, a robust CNN-based DCDD_Net was developed for correctly identifying chest diseases. Figure 4 illustrates the DCDD_Net model, which contains five convolutional blocks with rectified linear unit (ReLU) activation functions, a max pooling 2D layer, LecunUniform V2 as the kernel initializer, two dense layers, one dropout layer, and a SoftMax classification layer. Table 5 discusses the full structure of the network and the model summary of the proposed DCDD_Net for categorizing with the subsequent layer. The following subsections provide a brief description of the proposed model’s primary components.

#### 3.5.2. Proposed DCDD_Net Convolutional Blocks

The basic building block of the DCDD_Net that is being proposed is the convolutional block. A convolutional 2D layer, a ReLU layer, and a max-pooling 2D layer are included in each one of the convolutional blocks. To select weights for the convolutional 2D layer, the kernel initializer known as LecunUniform V2 is utilized. The gradient vanishing issue is addressed by utilizing the ReLU activation function, which also serves to boost the network’s capacity for learning and carrying out tasks. Concurrently, the convolutional 2D layer reduces the image and its dimensions in space by calculating the highest possible value throughout an input window (whose size is specified by the pool size) for all input channels. This layer operates randomly, and the features are increasingly constructed. In the initial layers, local patterns such as borders, lines, and shapes are taken out and local features are recovered based on those patterns. The model takes low-level, intermediate-level, and advanced features, allowing the deep model to accurately classify an image.

An input image of 128 × 128 × 3 was applied to the convolutional layer of block 1. The max pooling layer was used, which reduced the image size to 64 × 64 × 3. The ReLu function introduced non-linearity into the network’s computations, allowing it to learn and represent complex relationships in the CXR, CT scans, and cough sound image data. The same process was applied from block 2 to block 5. After that, the resultant feature vector was 8 × 8 × 128.

#### 3.5.3. Dropout Layer

After block 5, the dropout layer was placed. The dropout layer flips units on and off to lower network complexity and reduce model training time. To prevent models from overfitting, the dropout layer was set up to deactivate units on their own, according to a probability distribution, at the end of each epoch. As a consequence, the model obtained various features with each iteration as it discovered all relevant characteristics.

#### 3.5.4. Flatten Layer

This layer comes after the convolution layer and before the dense layer. In contrast to dense layers, convolution layers take tensor data forms as input, and only one-dimensional data forms are allowed in dense layers. The flattened layer was utilized to convert the 2D image representation into a 1D input.

### 3.6. Dense Blocks

The proposed DCDD_Net is made up of two dense layers, the details and the remaining layers of which will be discussed in the section that follows.

#### 3.6.1. ReLU Activation

The activation functions of a perceptron are computational functions that determine how the output of the perceptron should be conveyed to the layer that comes after it. In simple terms, they activate and terminate model nodes. The activation of ReLU is accomplished by exchanging every negative result with a value of zero. This activation function was applied to the convolutional layer outputs. In the output layer, the activation function is used to start the node that provides its label, which is subsequently allocated to the image that has been processed by the model. Multiple activation functions exist, but we implemented ReLU within hidden layers due to its basic and time-saving processing.

#### 3.6.2. Dense Layer

The dense layer accepts a single array as input and generates an output according to its parameters. This layer is also known as the fully connected layer. Images are recognized and assigned a label for their category within these layers. Using the back-propagation technique, the model learns in layers with complete connectivity. The number of parameters that can be trained on a model is defined by the number of different values that are employed in each dense layer. The final output of the model is generated by a SoftMax activation function, which classifies the image into one of the nine chest disease classes: COVID-19, normal, PNEUTH, ATE, EDE, COL, LC, TB, or PNEU. After a few layers, SoftMax is applied; it is a probability-based activation function in which the entire number of categories corresponds to the number of neurons [78].

### 3.7. Evaluation of the Proposed DCDD_Net

A confusion matrix is utilized to evaluate and compute the distinct metrics of a classification model. It contains the division of numbers and all of the estimations generated by a model throughout its testing and training steps. We employed multiple kinds of metrics to assess the effectiveness of the model. The efficiency of the proposed DCDD_Net for chest disease detection is typically measured using the following evaluation metrics (see Equations (2)–(7)):

(2) $$Accuracy=\frac{TP+TF}{TP+FN+FP+TN}$$

(3) $$Precision=\frac{TP}{TP+FP\;}$$

(4) $$Recall=\frac{TP}{TP+FN}$$

(5) $$F1-score=2\times \frac{Precision\times Recall}{Precision+Recall}$$

(6) $$TPR=\frac{FP}{FP+FN}$$

(7) $$FPR=\frac{FP}{FP+TN}$$

## 4. Results and Discussion

In the following section, we contrast DCDD_Net with the most recent deep networks. This section describes the distinctions between the proposed DCDD_Net and the four baseline deep networks.

### 4.1. Experimental Setup and Fine-Tuning of Hyperparameters

TensorFlow (TF) v. 2.12.0 was used to build the suggested model, whereas TF v. 1.8 was used to implement the four DL models (DenseNet-20, EfficientNet-B0, InceptionResNet-V2, and Xception). Furthermore, Python 3.10.1 was used to create methods that were not immediately related to convolutional networks. A PC with Windows 10 OS, 32 GB of RAM, and an 11 GB NVIDIA GPU was used for the experiment.

The DCDD_Net model utilizes imaging data from CXRs, CT scans, and cough sound images to identify nine distinct chest disease types. Grid search was used to optimize the performance of the DCDD_Net model by adjusting its hyperparameters (epoch, batch size, and learning rate). The DCDD_Net model was trained with a batch size of 32 and up to 50 testing epochs. The learning rates of the DCDD_Net model and the four DL models (DenseNet-20, EfficientNet-B0, InceptionResNet-V2, and Xception) were initially adjusted to 0.05 using the stochastic gradient descent (SGD) optimizer. We decreased the learning rate parameter by 0.1 when training showed no progress after 20 epochs. This was done to prevent the DCDD_Net model and the other four models from overfitting the data.

### 4.2. Accuracy of Proposed DCDD_Net with Baseline Models

**Figure 5:**
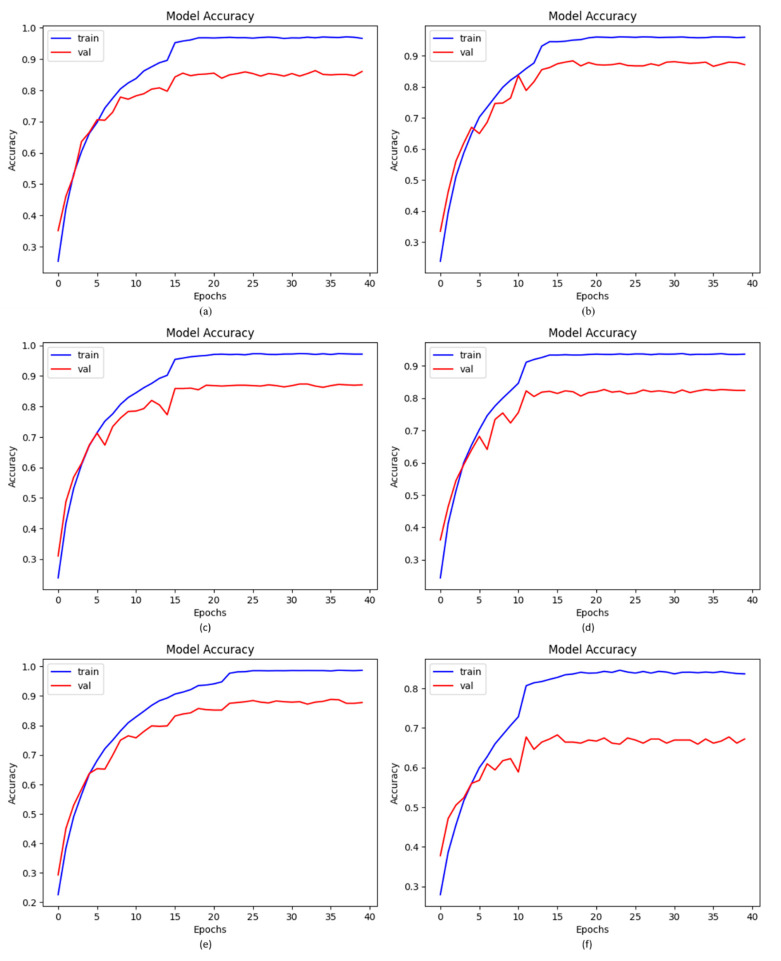
The remarkable enhancement in accuracy with or without BL-SMOTE in the proposed model with four networks: (**a**) DenseNet-201, (**b**) EfficientNet-B0, (**c**) InceptionResNet-V2, (**d**) Xception, (**e**) proposed model with BL-SMOTE, and (**f**) proposed model without BL-SMOTE.

By applying the same dataset both before and after balancing it using BL-SMOTE, we tested our proposed model and four baseline models, including DenseNet-201, EfficientNet-B0, InceptionResNet-V2, and Xception. For the suggested model, the BL-SMOTE technique presented remarkable outcomes. As shown in Table 6, the acquired accuracies for the suggested DCDD_Net models with and without BL-SMOTE, DenseNet-201, EfficientNet-B0, InceptionResNet-V2, and Xception were 96.67%, 66.15%, 85.37%, 86.04%, 87.25%, and 83.09%, respectively. Figure 5 shows the significant change gained by the suggested DCDD_Net model using BL-SMOTE.

### 4.3. Precision of Proposed DCDD_Net with Baseline Models

**Figure 6:**
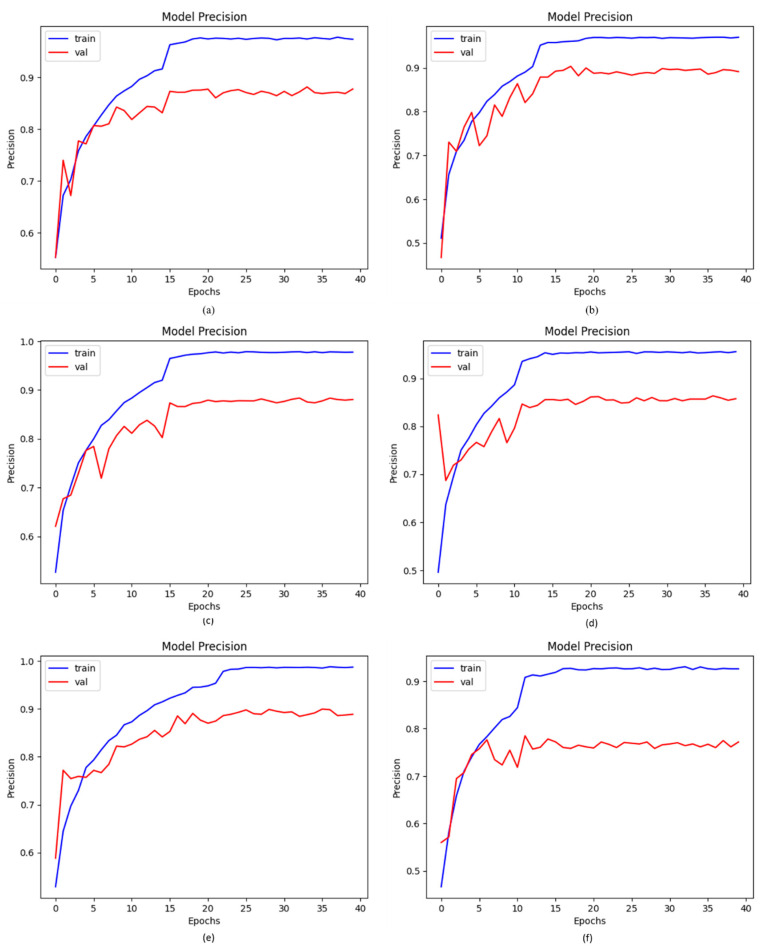
The remarkable enhancement in precision values with or without BL-SMOTE in the proposed model with four networks: (**a**) DenseNet-201, (**b**) EfficientNet-B0, (**c**) InceptionResNet-V2, (**d**) Xception, (**e**) proposed model with BL-SMOTE, and (**f**) proposed model without BL-SMOTE.

The proportion of accurate positive estimates to all positive predictions is known as precision. Using BL-SMOTE to equalize the dataset, we analyzed our proposed and existing networks, including DenseNet-201, EfficientNet-B0, InceptionResNet-V2, and Xception. For the suggested model, the BL-SMOTE technique presented remarkable outcomes. By applying the same dataset, the obtained precision values for the suggested DCDD_Net models with BL-SMOTE, without BL-SMOTE, DenseNet-201, EfficientNet-B0, InceptionResNet-V2, and Xception were 96.82%, 75.17%, 87.85%, 87.60%, 88.45%, and 85.78%, respectively. The study revealed that, in comparison to the four deep networks, the precision performance with BL-SMOTE of the proposed DCDD_Net was better and more reliable, as shown in Figure 6.

### 4.4. AUC of Proposed DCDD_Net with Other Networks

**Figure 7:**
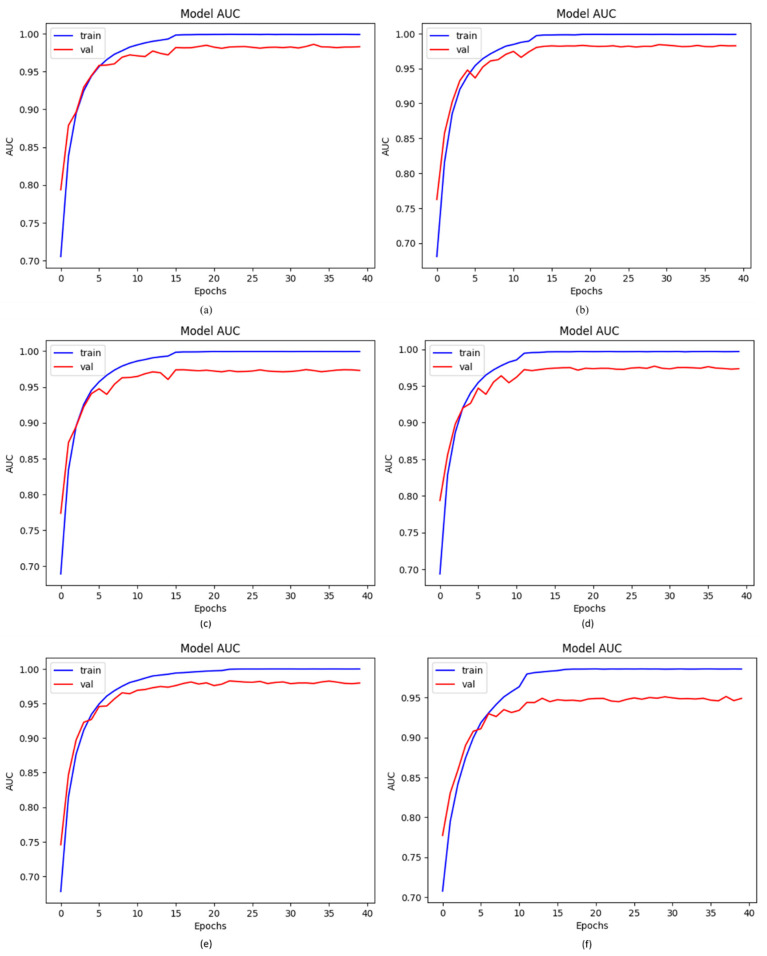
Significant improvement in values of AUC with or without BL-SMOTE in the proposed model with four networks: (**a**) DenseNet-201, (**b**) EfficientNet-B0, (**c**) InceptionResNet-V2, (**d**) Xception, (**e**) proposed model with BL-SMOTE, and (**f**) proposed model without BL-SMOTE.

As previously stated in this paper, our suggested model is a deep CNN-based DCDD-Net made up of several blocks that are particularly good at identifying the various kinds of chest diseases. To validate our deep DCDD-Net, we compared it to four other deep networks: DenseNet-201, EfficientNet-B0, InceptionResNet-V2, and Xception. The four baseline networks, DenseNet-201, EfficientNet-B0, InceptionResNet-V2, and Xception, acquired AUC values of 98.22%, 97.75%, 97.98%, and 97.90%, respectively. Figure 7 shows that the proposed DCDD_Net with BL-SMOTE and DCDD_Net without BL-SMOTE, after employing the datasets, achieved 99.43% and 95.31% AUC values, respectively. We concluded that the suggested model’s AUC findings continued to outperform those of other models based on the previous evaluation.

### 4.5. Recall of Proposed DCDD_Net with Other Networks

**Figure 8:**
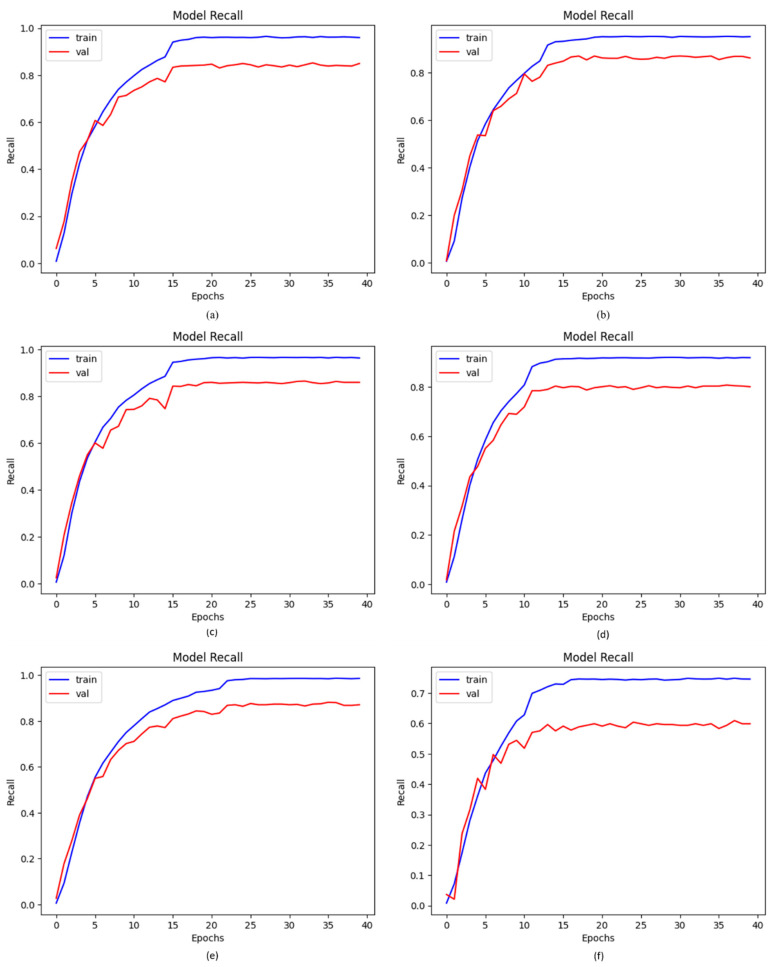
Notable enhancement in values of recall with or without BL-SMOTE in the proposed model with four networks: (**a**) DenseNet-201, (**b**) EfficientNet-B0, (**c**) InceptionResNet-V2, (**d**) Xception, (**e**) proposed model with BL-SMOTE, and (**f**) proposed model without BL-SMOTE.

Based on the recall measure, the ability of the model to recognize positive samples was assessed. The values of recall that were high demonstrate that more positive samples were found. Recall curves were employed to evaluate the proposed DCDD_Net with four baseline networks, as shown in Figure 8. The proposed DCDD_Net with and without BL-SMOTE, DenseNet-201, EfficientNet-B0, InceptionResNet-V2, and Xception produced recall values of 95.76%, 58.66%, 84.43%, 84.43%, 86.31%, and 80.94%, respectively. The proposed technique showed impressive recall performance as a result of the stated explanation.

### 4.6. F1-Score of Proposed DCDD_Net with Other Networks

**Figure 9:**
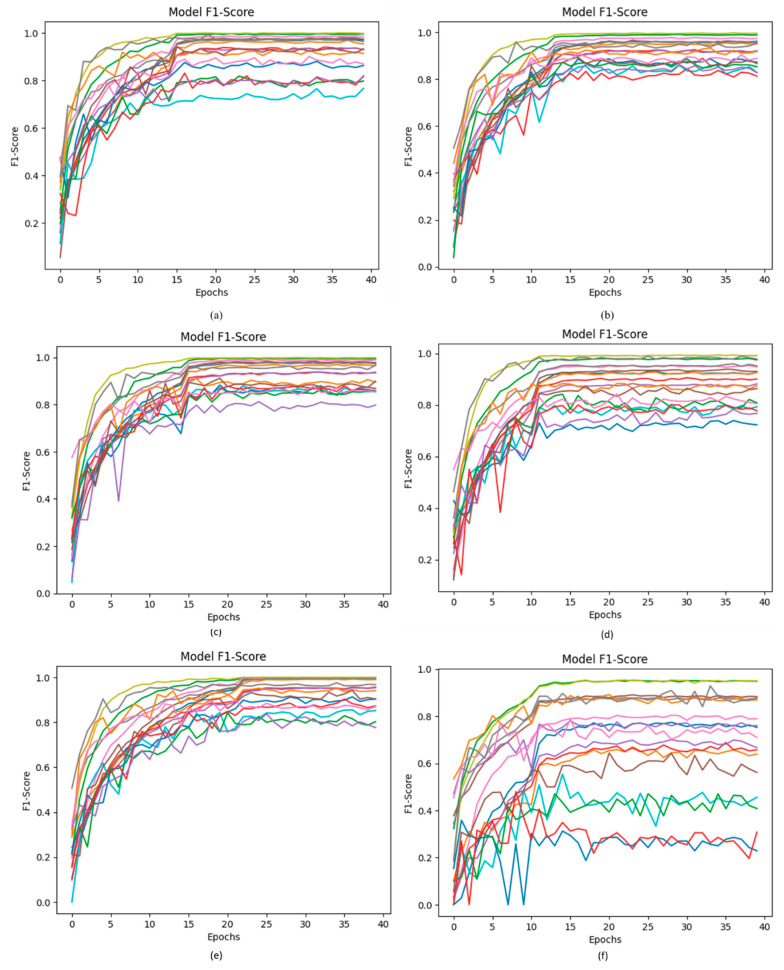
Computation of the values of the F1-score among the proposed DCDD_Net and four baseline networks: (**a**) DenseNet-201, (**b**) EfficientNet-B0, (**c**) InceptionResNet-V2, (**d**) Xception, (**e**) proposed model with BL-SMOTE, and (**f**) proposed model without BL-SMOTE.

In this proposed DCDD-Net model, the input dataset is normalized, and the one-hot encoder is fundamentally used for adding categorical data variables to the model. The uneven dataset issue is subsequently resolved using the BL-SMOTE technique by oversampling the categories to equalize the dataset. Figure 9 illustrates the significant increase in the F1-score of the proposed DCDD-Net using BL-SMOTE. The proposed DCDD-Net with BL-SMOTE, DCDD-Net without BL-SMOTE, DenseNet-201, EfficientNet-B0, InceptionResNet-V2, and Xception obtained F1-score values of 95.61%, 55.48%, 84.88%, 85.79%, 87.04%, and 82.88%, respectively, as shown in Figure 9.

### 4.7. Loss of Proposed DCDD_Net with Other Networks

**Figure 10:**
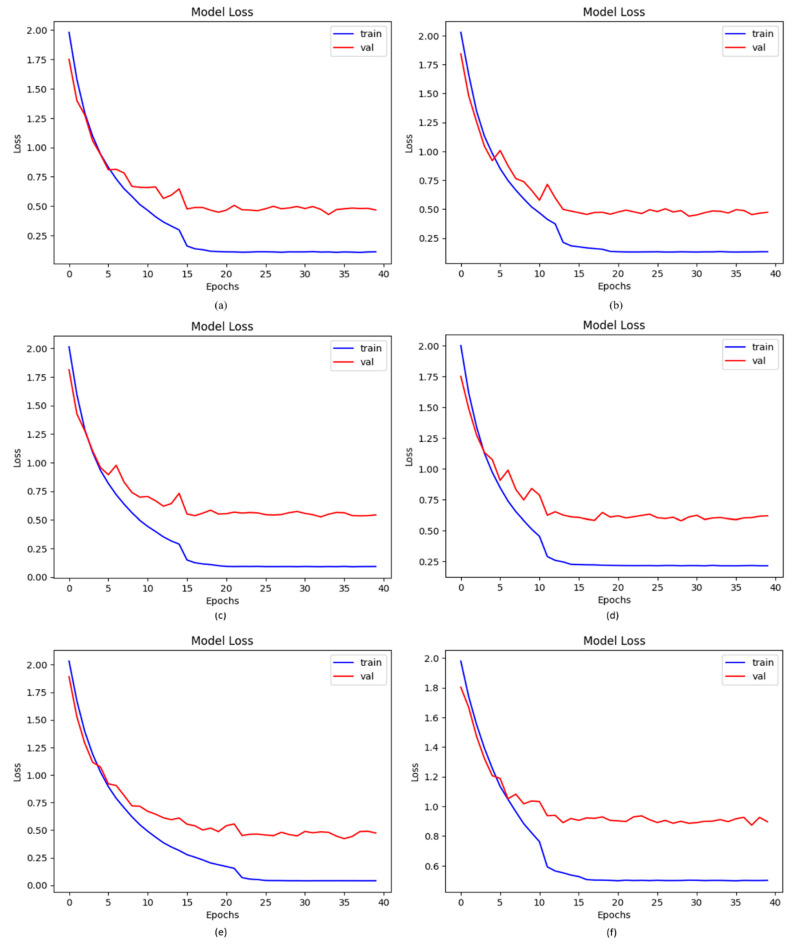
Computation of the loss values among the proposed DCDD_Net and four baseline networks: (**a**) DenseNet-201, (**b**) EfficientNet-B0, (**c**) InceptionResNet-V2, (**d**) Xception, (**e**) proposed model with BL-SMOTE, and (**f**) proposed model without BL-SMOTE.

The numerical difference between the expected and actual values is calculated via loss functions. The loss in this study was determined using a categorical cross-entropy technique. However, the results were even more impressive when the model was developed on upsampled images. The suggested DCDD_Net with and without BL-SMOTE produced loss values of 0.1477 and 0.8732, respectively, whereas DenseNet-201, EfficientNet-B0, InceptionResNet-V2, and Xception acquired loss values of 0.4638, 0.5153, 0.5122, and 0.5443, respectively. The suggested DCDD_Net system with BL-SMOTE’s notable reduction in loss value is shown in Figure 10.

### 4.8. ROC of Proposed DCDD_Net with Other Networks

**Figure 11:**
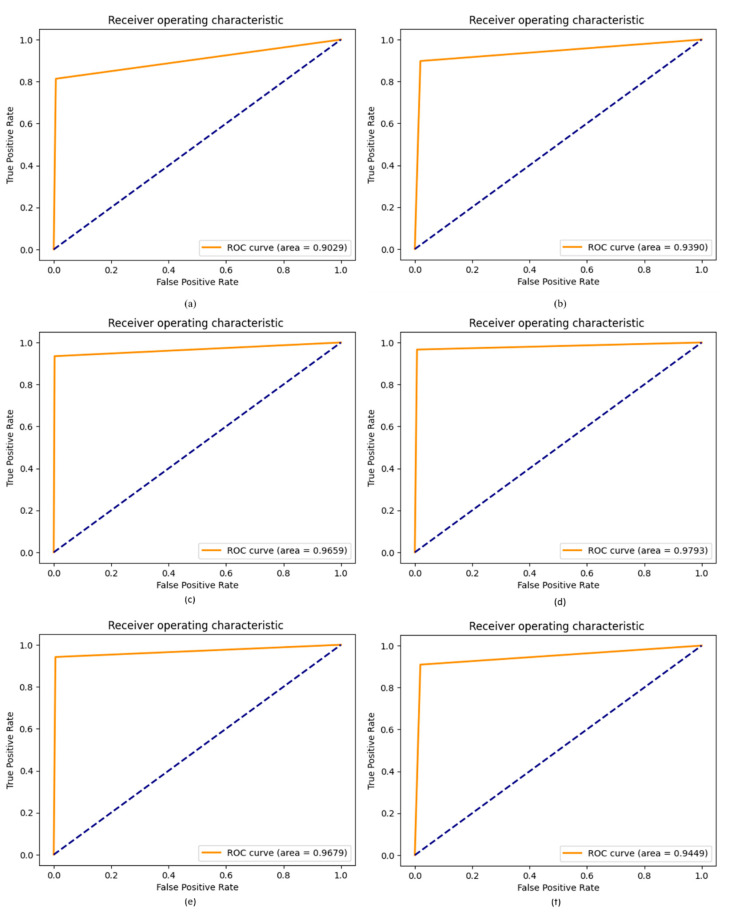
Substantial enhancement in values of the ROC curve among the proposed model with four networks: (**a**) DenseNet-201, (**b**) EfficientNet-B0, (**c**) InceptionResNet-V2, (**d**) Xception, (**e**) proposed model with BL-SMOTE, and (**f**) proposed model without BL-SMOTE.

A ROC curve is employed to assess the effectiveness of an algorithm for binary or multi-class classification and the results of a clinical examination. The effectiveness of the classifier is measured using the area under the curve (AUC) on an ROC curve, where a greater AUC often indicates a more useful classifier. By employing the same dataset with and without BL-SMOTE, we used the ROC curve to evaluate the effectiveness and accuracy of our suggested DCDD-Net. Figure 11 shows the ROC values for the proposed DCDD-Net with BL-SMOTE and DCDD-Net without BL-SMOTE.

### 4.9. ROC Extension of Proposed DCDD_Net with Other Networks

**Figure 12:**
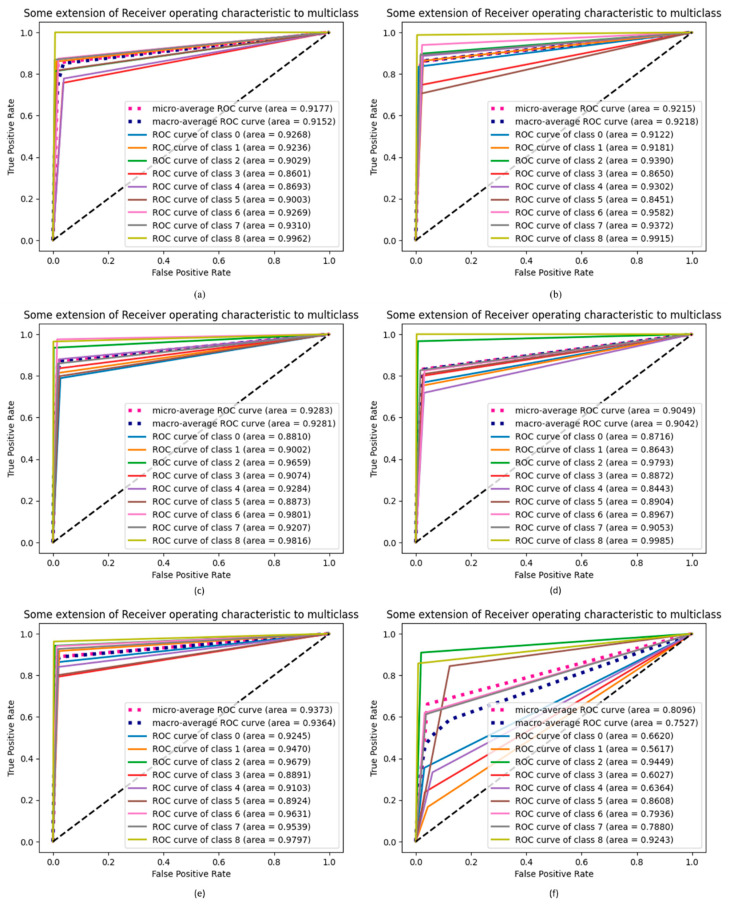
Computation of the extension of the ROC among the proposed DCDD_Net and four baseline networks: (**a**) DenseNet-201, (**b**) EfficientNet-B0, (**c**) InceptionResNet-V2, (**d**) Xception, (**e**) proposed model with BL-SMOTE, and (**f**) proposed model without BL-SMOTE.

Figure 12 compares the proposed DCDD-Net with DenseNet-201, EfficientNet-B0, InceptionResNet-V2, and Xception, utilizing the extension of the ROC curve. As can be seen in Figure 12, the AUC for the proposed techniques was greatly increased compared to that of other networks after the dataset was balanced by the BL-SMOTE technique. The proposed DCDD-Net with BL-SMOTE and DCDD-Net without BL-SMOTE for classes 0 (atelectasis), 1 (consolidation lung), 2 (COVID_19), 3 (edema), 4 (lung cancer), 5 (normal), 6 (pneumonia), 7 (pneumothorax), and 8 (tuberculosis) both showed a similar effect. The enhancements in AUC demonstrate the reliability of the BL-SMOTE method and DCDD-Net feature selection.

### 4.10. Confusion Matrix of Proposed DCDD_Net with Baseline Models

**Figure 13:**
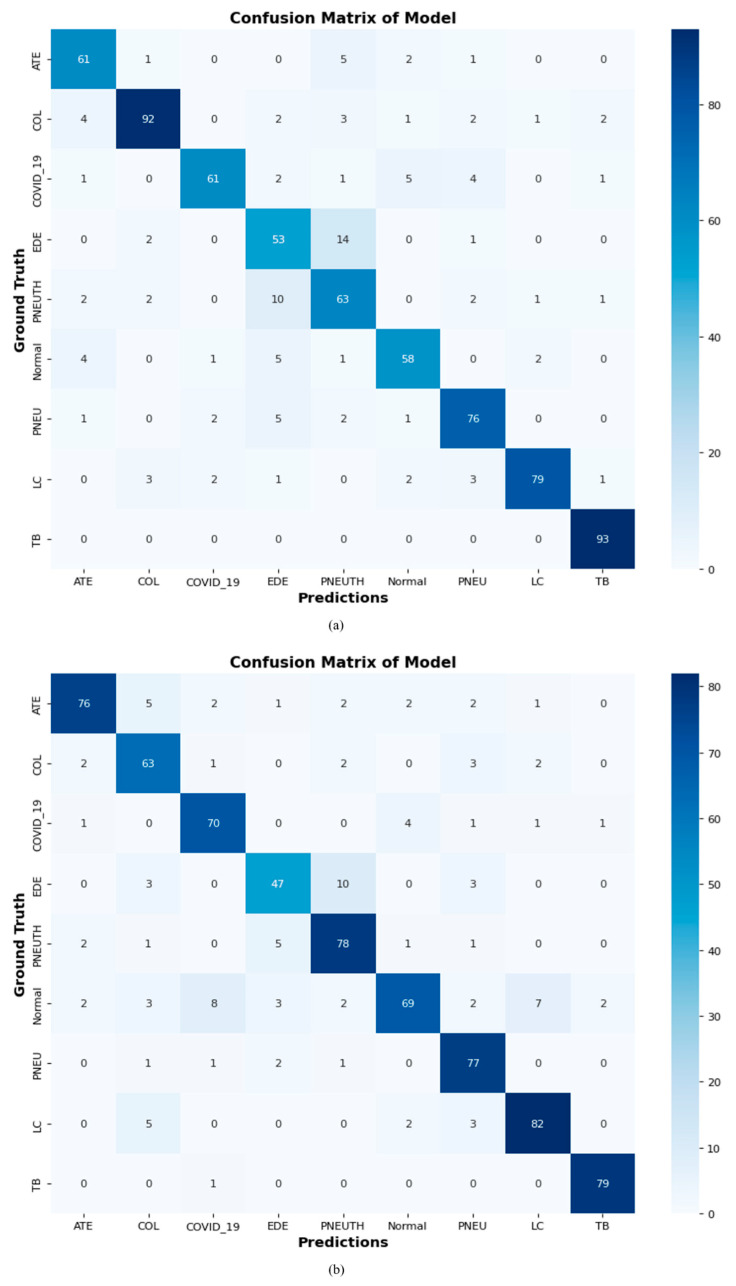

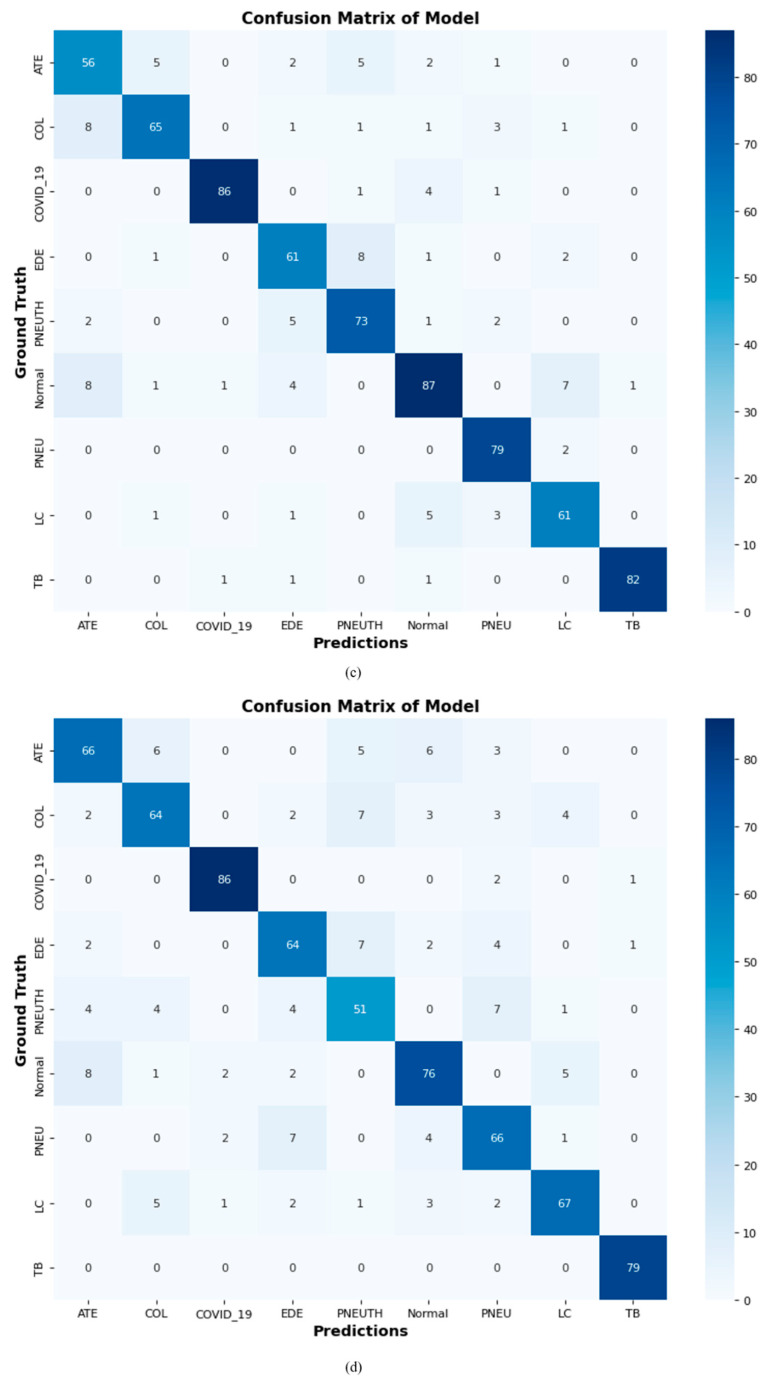

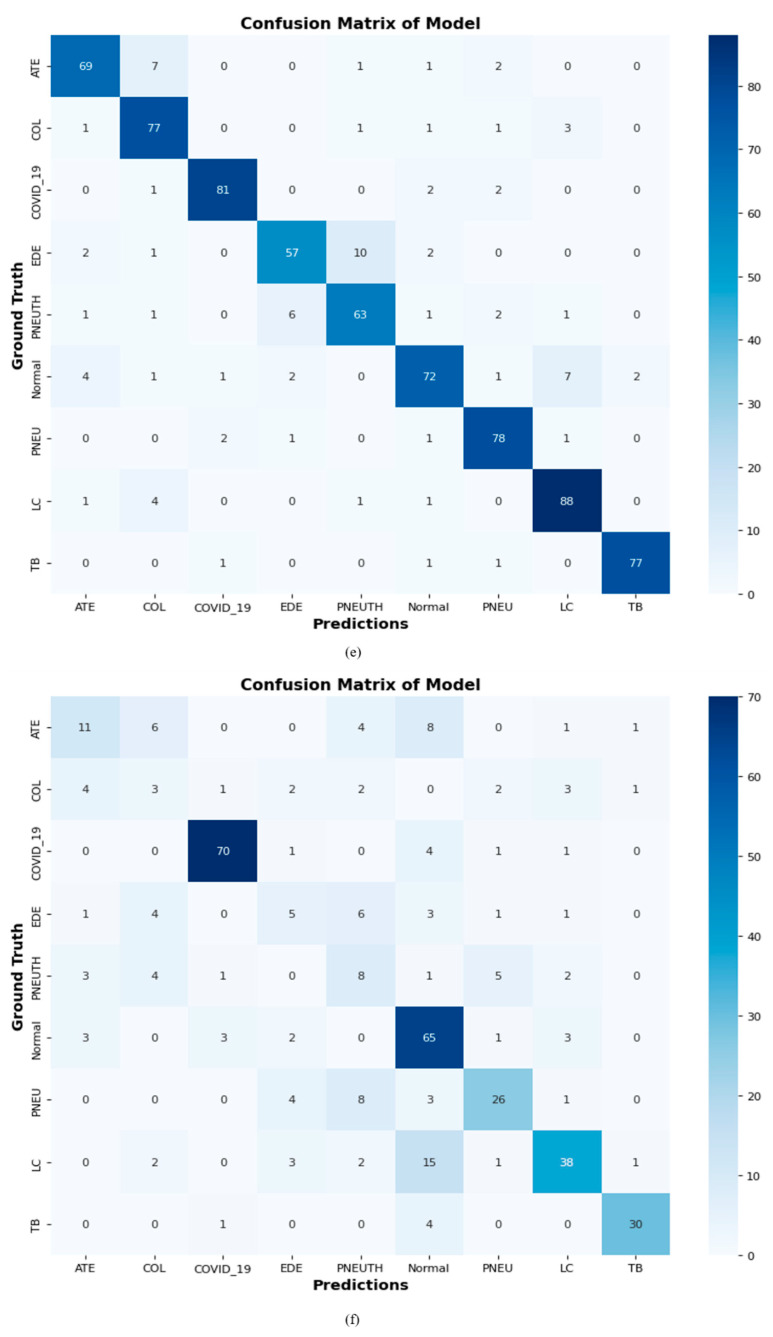
Employing a confusion matrix to compare the proposed DCDD_Net and four networks: (**a**) DenseNet−201, (**b**) EfficientNet−B0, (**c**) InceptionResNet−V2, (**d**) Xception, (**e**) proposed model with BL−SMOTE, and (**f**) proposed model without BL-SMOTE.

We analyzed our proposed DCDD_Net model with four other networks to verify it with a confusion matrix. The DCDD_Net model greatly improved with the implementation of BL-SMOTE, as seen in Figure 13.

### 4.11. Statistical Analysis

Comparisons were made between the proposed model and the base classifiers, whose probability scores were used to determine the proposed model’s construction using the McNemar test [79] and the analysis of variance (ANOVA) test [80]. The McNemar and ANOVA tests were run on the multi-chest disease datasets of CXR, CT scans, and cough sound images, and the results are shown in Table 7. Both the McNemar and the ANOVA test require a smaller *p*-value (i.e., 0.05) to reject the null hypothesis. Table 7 demonstrates that all sample *p*-values were significantly smaller than 0.05. The results of both statistical tests contradicted the null hypothesis. This demonstrates that the suggested model was statistically distinct from the other contributing models since it combined more information from the base classifiers and produced better predictions.

### 4.12. Comparison of the Proposed DCDD_NET Using State-of-the-Art

In this section, we evaluate the suggested DCDD_Net model with previous research [82,83,84,85,86,87]. In comparison to prior SOTA studies, Table 8 provides an in-depth analysis of the proposed DCDD_Net model in the context of numerous performance assessment criteria, including accuracy, recall, and F1-score.

### 4.13. Discussion

In the present work, a CNN-based DCDD_Net model is proposed for chest disease detection. Our DCDD_Net model showed remarkable categorization in the domains of EDE, normal, COL, COVID-19, PNEU, PNEUTH, LC, TB, and ATE compared to the classification performance of the other four deep networks. On datasets with a fixed image resolution of 128 × 128 × 3, our DCDD_Net model and four baseline networks, including DenseNet-201, EfficientNet-B0, InceptionResNet-V2, and Xception, were trained. In this study, three types of datasets were used: CXR [40], CT scan [41], and cough sounds [42] of chest diseases. Radiologists frequently employ CXR imaging to quickly and affordably diagnose a variety of bodily organs, including the heart, bones, blood vessels, lungs, and airways. This is crucial for identifying illnesses and anomalies. X-ray radiation is often projected into the body while laying on the metallic plate of the X-ray equipment to produce CXR images.

A CT scan is a medical diagnostic process that creates images of the chest using an integration of X-rays and computer technology. Cross-sectional images are produced using a CT scan, which combines several X-ray images collected at various angles. Scalograms represent the actual frequencies of a wave’s continuous wavelet transform (CWT) factors [82,83,84,85,86,87]. Cough signals utilize CWT to convey data from the time domain to the frequency domain, as demonstrated in Figure 3. The chest disease dataset was categorized into three separate groups: testing, training, and validation. In addition, Figure 1 illustrates the workflow of the proposed DCDD_Net for the identification of chest diseases.

To tackle the problem of unequal class representation in the dataset, we referred to the upsampling methodology. In this method, the classification process begins with the analysis of the minority class. Table 2 depicts the arrangement of samples before the start of the upsampling process. The order in which the samples were distributed can be seen in Table 3, which was generated after the upsampling was performed. As shown in Table 5, the acquired accuracies for the suggested DCDD_Net models with and without BL-SMOTE, DenseNet-201, EfficientNet-B0, InceptionResNet-V2, and Xception were 96.67%, 66.15%, 85.37%, 86.04%, 87.25%, and 83.09%, respectively. The DCDD_Net model, which includes a SoftMax classification layer, two dense layers, one dropout layer, a max pooling layer in 2D, and five convolutional blocks with rectified linear unit (ReLU) activation functions, is shown in Figure 4. The whole network layout and the model overview of the suggested DCDD_Net for layer-following categorization are covered in Table 4. The examination of the experimental data shows that the multi-classification of chest disorders using the CXR, CT scan, and cough sound added a considerable and useful output to aid human diagnosticians.

The success rate for the classification of the proposed DCDD_Net with SOTA classifiers is shown in Table 8. Ibrahim et al. [82] used the AlexNet model for the classification of five chest diseases using CXR images. They obtained the images from different public databases. Constantinou et al. [83] used ResNet101, DenseNet121, ResNet50, InceptionV3, and DenseNet169 for the detection of COVID-19. All models performed effectively, but ResNet101 outperformed the others, scoring 96% in precision, accuracy, and recall. Malik et al. [84] developed a CDC_Net model to automatically identify COVID-19, PNEUTH, TB, LC, and PNEU from CXR images. They achieved 90.39% accuracy, a recall of 90.13%, and 92.26% precision. A framework for the automatic detection of COVID-19 employing chest CT scan pictures and DL-based algorithms was developed by Gupta et al. [85]. Using DarkNet 19, the greatest accuracy in classification of 94.91% was obtained.

## 5. Conclusions

In the current study, a multi-classification DCDD_Net model for identifying nine chest diseases from CXR, CT scan pictures, and cough sounds was developed. Chest diseases represent some of the most prevalent health issues in the world; they are possibly fatal diseases that may impact organs, including the heart and lungs. An extremely large number of cases demands a rapid and effective diagnostic procedure. Due to incorrect and ineffective testing procedures, poor facilities, and the inability to recognize various chest diseases at an early stage, many people have passed away and been taken to ICUs. We developed a technique that identifies nine chest diseases, including EDE, normal, COL, COVID-19, PNEU, PNEUTH, LC, TB, and ATE. The modified structure’s convolutional blocks were created using numerous layers and used to categorize early-stage chest diseases. To overcome dataset imbalance issues and keep the number of images for each class in balance, images were created using the BL-SMOTE algorithm. Our proposed DCDD_Net model obtained a 99.43% AUC, a 95.61% F1-score, 95.76% recall, 96.82% precision, and 96.67% accuracy. A comprehensive experiment indicated that, as compared to widely recognized pre-trained and cutting-edge classifiers, our suggested DCDD_Net performed the best in terms of diagnostic performance. The limitation of the study is that the proposed model is not suitable for identifying chest diseases from breath sounds and sonography images. In the future, we will integrate blockchain, a deep attention module, and federated learning to classify diseases of the chest more accurately.

## Figures and Tables

**Table 2:** Statistical information regarding coughing audio datasets.

Chest Diseases	No. of Cough Audios for Each Class	Total Audio in Minutes (m)	Standard Sounds per Person in Seconds (s)	Standard Deviation
COVID-19	100	32 (m)	2.77 (s)	1.61 (s)
EDE	39	32 (m)	2.05 (s)	1.04 (s)
Normal	210	120 (m)	3.92 (s)	1.79 (s)
COL	80	24 (m)	2.61 (s)	1.30 (s)
PNEU	119	57 (m)	2.02 (s)	1.01 (s)
PNEUTH	42	37 (m)	2.11 (s)	1.06 (s)
LC	222	60 (m)	2.15 (s)	1.07 (s)
TB	292	60(m)	3.12 (s)	1.61 (s)
ATE	90	27 (m)	2.52 (s)	1.24 (s)
Total	1194	449 (m)	23.27 (s)	11.73 (s)

**Table 3:** Distribution of chest disease image samples before BL_SMOTE.

No. of Classes	Class Name	CXR	CT Scan	Cough Sound	Total
0	COVID_19	423	426	100	949
1	Normal	247	672	210	1129
2	TB	259	112	292	663
3	PNEU	189	168	119	476
4	LC	174	118	222	515
5	PNEUTH	425	160	42	627
6	ATE	154	217	90	461
7	COL	154	112	80	346
8	EDE	198	91	39	328

**Table 4:** Distribution of chest disease image samples after BL_SMOTE.

No. of Classes	Class Name	Total	Training (70%)	Validation (20%)	Testing (10%)
0	COVID_19	1129	790	225	114
1	Normal	1129	790	225	114
2	TB	1129	790	225	114
3	PNEU	1129	790	225	114
4	LC	1129	790	225	114
5	PNEUTH	1129	790	225	114
6	ATE	1129	790	225	114
7	COL	1129	790	225	114
8	EDE	1129	790	225	114

**Table 5:** List of parameters applied in the proposed DCDD_Net.

Layer Type	Output Shape	Parameters
Input layer	(None, 128, 128, 3)	0
Block 1	(None, 128, 128,8)	224
Block 2	(None, 64, 64, 16)	1168
Block 3	(None, 32, 32, 32)	4640
Block 4	(None, 16, 16, 64)	18,496
Block 5	(None, 8, 8, 128)	73,856
Dropout layer	(None, 4, 4, 128)	0
Flatten	(None, 2048)	0
Dense block 1	(None, 512)	1,049,088
Dense layer	(None, 9)	4617
Output: SoftMax	(None, 9)	0
Total parameters:		1,152,089
Trainable parameters:		1,152,089
Non-trainable parameters:		0

**Table 6:** DCDD_Net model’s performance with four baseline networks.

Classifiers	Accuracy	Precision	Recall	F1-Score	AUC	Trainable Parameters
DenseNet-201	85.37%	87.85%	84.42%	84.88%	98.22%	5,431,999
EfficientNet-B0	86.04%	87.60%	84.43%	85.79%	97.75%	4,587,852
InceptionResNet-V2	87.25%	88.45%	86.31%	87.04%	97.98%	6,123,027
Xception	83.09%	85.78%	80.94%	82.88%	97.90%	5,965,411 S
Proposed model (with BL-SMOTE)	96.67%	96.82%	95.76%	95.61%	99.43%	1,152,089
Proposed model (without BL-SMOTE)	66.15%	75.17%	58.66%	55.48%	95.31%	2,263,190

**Table 7:** Results of the McNemar and ANOVA tests on the DCDD_Net model.

Sr#	Statistical Analyses	Outcomes
1	McNemar test	0.0140
2	ANOVA test	0.0011

**Table 8:** Comparison of the DCDD_Net model with recent SOTA.

Reference	Year	Model	Diagnostic Technique	Accuracy	Recall	F1-Score
[82]	2021	AlexNet	CXR	94.00%	91.30%	-
[83]	2023	ResNet101, DenseNet121, ResNet50, InceptionV3, and DenseNet169	CXR	92.00%	91.00%	90.00%
[84]	2022	CDC_Net	CXR	90.39%	90.13%	92.26%
[85]	2023	DarkNet19	CT scan	94.91%	93.96%	94.52%
[86]	2023	SVM	CT scan	95.90%	-	-
[87]	2022	MSCCov19Net	Cough sound	90.40%	-	-
Ours	-	DCDD_Net with BL-SMOTE	CXR, CT scan, and cough sound/images	96.67%	95.61%	99.43%

## Data Availability

All datasets used in this study are benchmark and publicly available.
